# Disparities in mortality‐to‐incidence ratios by race/ethnicity for female breast cancer in New York City, 2002‐2016

**DOI:** 10.1002/cam4.3309

**Published:** 2020-10-02

**Authors:** Tamar B. Nobel, Charles K. Asumeng, John Jasek, Kellie C. Van Beck, Ruchi Mathur, Baozhen Qiao, Jennifer J. Brown

**Affiliations:** ^1^ Department of Environmental Medicine and Public Health Mount Sinai Hospital New York NY USA; ^2^ Cancer Prevention and Control Program Bureau of Chronic Disease Prevention New York City Department of Health and Mental Hygiene Queens NY 11101 USA; ^3^ Research and Evaluation, Bureau of Chronic Disease Prevention NYC DOHMH Queens NY USA; ^4^ Bureau of Cancer Epidemiology New York State Department of Health Albany NY USA

**Keywords:** breast cancer, breast cancer mortality rate, breast cancer stage, racial disparities, New York City

## Abstract

**Background:**

Racial disparities in New York City (NYC) breast cancer incidence and mortality rates have previously been demonstrated. Disease stage at diagnosis and mortality‐to‐incidence ratio (MIR) may present better measures of differences in screening and treatment access. Racial/ethnic trends in NYC MIR have not previously been assessed.

**Methods:**

Mammogram rates were compared using the NYC Community Health Survey, 2002‐2014. Breast cancer diagnosis, stage, and mortality were from the New York State Cancer Registry, 2000‐2016. Primary outcomes were MIR, the ratio of age‐adjusted mortality to incidence rates, and stage at diagnosis. Joinpoint regression analysis identified significant trends.

**Results:**

Mammogram rates in 2002‐2014 among Black and Latina women ages 40 and older (79.9% and 78.4%, respectively) were stable and higher than among White (73.6%) and Asian/Pacific‐Islander women (70.4%) (*P* < .0001). There were 82 733 incident cases of breast cancer and 16 225 deaths in 2000‐2016. White women had the highest incidence, however, rates among Black, Latina, and Asian/Pacific Islander women significantly increased. Black and Latina women presented with local disease (Stage I) less frequently (53.2%, 57.6%, respectively) than White (62.5%) and Asian/Pacific‐Islander women (63.0%). Black women presented with distant disease (Stage IV) more frequently than all other groups (Black 8.7%, Latina 5.8%, White 6.0%, and Asian 4.2%). Black women had the highest breast cancer mortality rate and MIR (Black 0.25, Latina 0.18, White 0.17, and Asian women 0.11).

**Conclusions:**

More advanced disease at diagnosis coupled with a slower decrease in breast cancer mortality among Black and Latina women may partially explain persistent disparities in MIR especially prominent among Black women. Assessment of racial/ethnic differences in screening quality and access to high‐quality treatment may help identify areas for targeted interventions to improve equity in breast cancer outcomes.

## Graphical abstract

Lower incidence of local stage breast cancer at diagnosis and slower decrease in mortality rates among Black and Latina women in NYC as compared to White women raises great concern about unequal access to quality care including quality screening and improvements in therapy. These differences were especially prominent among Black women, who merit additional public health focus.

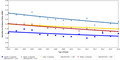

## 1 INTRODUCTION

Breast cancer is the most common malignancy among women in the US and the second most frequent cause of cancer‐related death.^1^ With improvements in screening and treatment, breast cancer mortality in the US has decreased by nearly 40% over the last 40 years.^2^ Given that stage at diagnosis is one of the most important predictors of breast cancer prognosis, it is imperative to identify the disease earlier while curative therapy can still be pursued.^3^ Recent data have demonstrated 5‐year overall survival is 98% for women with local tumors as compared to 27% for those with distant tumors at diagnosis.^2^ Since the implementation of the National Breast and Cervical Cancer Early Detection Program in 1990, which provides breast and cervical cancer early detection testing to low‐income, underserved, and under and un‐insured women in the US, there has been increased focus on ensuring access to breast cancer screening and treatment for low‐income and underserved women at the municipal, state, and federal levels.^4^, ^5^ Despite these efforts, well‐described national racial/ethnic disparities in breast cancer mortality suggest that advances in diagnosis and treatment may not be equally benefitting all women.^6^, ^7^, ^8^

In the US, White women have historically had a higher breast cancer incidence rate than Black women. Breast cancer mortality rates have been higher among Black women than White women. Mortality remains over 40% higher among Black women although recent data have suggested that this gap has narrowed.^9^, ^10^, ^11^ Cancer‐related mortality rates are an important measure of disparities; however, failure to account for disease‐related death relative to the incidence within a group may underestimate the meaning of observed trends.^12^ Given the observed differences in breast cancer incidence between racial/ethnic groups, the mortality‐to‐incidence ratio (MIR) may provide a better understanding of inequities in breast cancer prognosis in a large population such as New York City (NYC), where survival studies are less feasible and limited in size. MIR allows for a more standardized comparison of disease burden as defined by mortality, few studies use this parameter to evaluate racial/ethnic differences in female breast cancer in the US.^12^, ^13^ A limitation of MIR is that it cannot identify causative factors or mechanisms that lead to differences among groups. It is an indirect measure as the cases of mortality and incidence are not necessarily the same people.^14^ Also, breast cancers have a natural variation in incidence and mortality over the years that affects accuracy of MIR.

Disparities in breast cancer stage and mortality in NYC have previously been described but studies are limited by lack of inclusion of Latina and Asian/Pacific Islander women, who together comprise over 40% of the NYC population.^15^, ^16^, ^17^, ^18^, ^19^, ^20^, ^21^ Improved understanding of the current state of inequities in breast cancer for all women in NYC is needed.

To our knowledge, this is the first study to compare racial/ethnic breast cancer disparities in NYC using trends in MIR as the primary outcome measure, and to include Latina ethnicity and Asian/Pacific‐Islander race. We hypothesize that using MIR will enable better understanding of disparities by accounting for known racial/ethnic differences in this large urban population.

## 2 METHODS

### 2.1 Data source and study population

Mammography data were obtained from the New York City Community Health Survey (CHS), an annual telephone survey (dual‐frame landline and cell phone since 2009) conducted by the Department of Health and Mental Hygiene (DOHMH).^22^ The survey includes an annual sample of approximately 10 000 adults from all five boroughs. Weighted data are available for public access on EpiQuery.^23^ Age‐adjusted self‐reported mammography rates (within the past 2 years) among women age ≥ 40, the age recommended to discuss screening with a healthcare provider by national recommendations, by race/ethnic group were abstracted CHS from available years (2002, 2004‐2010, 2012, 2014). Due to periodic rotation of survey items, the mammography question was not included in the CHS in 2003, 2009, 2013, or after 2014 and so these data were not available.

Breast cancer incidence, mortality, and stage at diagnosis data, and poverty level for women of all ages were obtained from the New York State Cancer Registry (NYSCR).^24^ The NYSCR has collected cancer information in NYC from mandated reporting by physicians, dentists, laboratories, and other health care providers since 1976, with information on Latina ethnicity available since 2000. It is a participant of the North American Association of Central Cancer Registries certification process and consequently, the data are evaluated for timelines, completeness, and quality annually.

The study cohort included women of any age in the NYSCR database with breast cancer diagnosed between January 1, 2002 and December 31, 2016 in NYC. Analyses were stratified by four mutually exclusive racial/ethnic groups: Latinas (of any race), non‐Latina Whites, non‐Latina Blacks, and Asian/Pacific‐Islanders.

### 2.2 Breast cancer indicators

The primary outcome of interest was mortality‐to‐incidence ratio (MIR). MIR has previously been established as a high‐level comparative measure to identify inequities in cancer control programs and cancer survival by normalizing the disease‐specific mortality rate to the incidence of disease for a given group of interest.^12^, ^25^, ^26^ MIR was calculated as the mortality rate divided by incidence rate for a given year for each racial/ethnic group. Incidence included new unduplicated breast cancer diagnoses. Age‐adjusted rates were calculated per 100 000 women as per the 2000 US census population and stratified by race/ethnic group (White, Black, Latina, Asian/Pacific‐Islander). Incidence was further analyzed by the rate of incident cases with local and distant stage disease at diagnosis.

### 2.3 Statistical analysis

SEER*Stat version 8.3.5 was used to identify the breast cancer cohort and obtain incidence and mortality rates by race/ethnicity, year of diagnosis, and case listing of breast cancer diagnoses during the study period. Available demographics were compared by racial/ethnic group. Chi‐square analysis was used to evaluate differences between groups.

The Joinpoint regression analysis program was used to evaluate the data for significant linear trends. Joinpoint provides both the annual percent change (APC) and average annual percent change (AAPC), where APC is the rate of change over 1‐year period and AAPC is the weighted average over the identified trend segment. The software analyzes trend data to fit the simplest Joinpoint model that the data will allow, where zero joinpoints is a straight line. The analysis tests whether additional joinpoints are statistically significant and adds them to the model using Monte Carlo permutation.^27^ The direction and magnitude of the estimated trend are given. In describing the trends, the terms increase or decrease were used when the APC was statistically significant; otherwise, the term stable was used. Pairwise tests for parallelism between racial/ethnic groups were conducted to assess for differences in APC between racial/ethnic groups.

The disease stage at diagnosis was evaluated as the incidence rate of local (Stage I) and distant (Stage IV) disease at diagnosis as within the overall annual incident case rate by racial/ethnic group. ANOVA was used to test overall differences between mean annual incidence, the proportion with a given diagnostic stage and mortality by racial/ethnic group, using the Tukey method to adjust for multiple comparisons. CHS data were used to present the percentage of women who reported a mammography within the last 2 years by race/ethnicity, and the mean mammography rate across study years was calculated by racial/ethnic group using available data. SAS v9.4 (SAS Institute) and Joinpoint version 4.5.0.1 (National Cancer Institute) were used to perform statistical analysis. A *P* value less than .05 was considered statistically significant.

## 3 RESULTS

### 3.1 Mammogram rates and race/ethnicity

**FIGURE 1:**
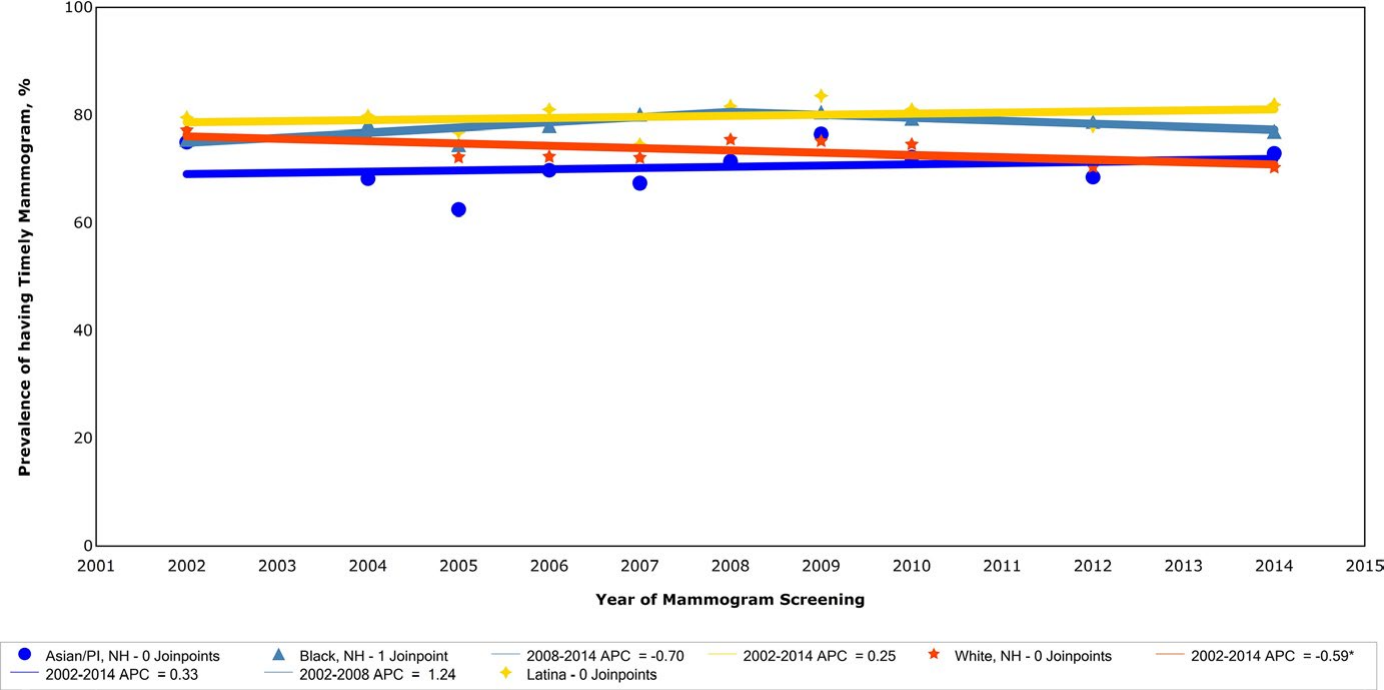
Mammography within the past 2 y among women ages 40 and older in NYC by race/ethnicity (age‐adjusted), 2002‐2014. Trends in age‐adjusted rates of mammography within 2 y for NYC women ages 40 and older by race/ethnicity, from the NYC CHS, 2002‐2014. CHS, NYC Community Health Survey; NH, non‐hispanic; NYC, New York City

Age‐adjusted rates of self‐reported mammography within the last 2 years by race/ethnicity group and CHS year are presented in Figure 1. Mammography rates of Black, Latina and Asian women were relatively stable across the time period of 2002 to 2014 with no significant changes. Mammography rates among White women declined slightly by 0.59% per year from 2002 to 2014. The mean rates across all years among Black and Latina women (79.9% and 78.4%, respectively) were significantly higher than among White (73.6%) and Asian/Pacific‐Islander women (70.4%) (*P* < .0001) (Figure 1).

### 3.2 Incidence

There were 82 733 incident cases of breast cancer diagnosed between 2002 and 2016 in NYC and included in our analysis. Characteristics of the included population are presented in Table 1. The greatest proportion of cases were among White women compared with other groups (47.6%). Black, Latina, and Asian/Pacific Islander women with breast cancer were more likely to be younger, foreign born, and living in higher poverty neighborhoods than White women (*P* < .0001).

**TABLE 1:** Characteristics of women diagnosed with breast cancer in NYC, 2002‐2016

Characteristic	White, NH	Black, NH	Asian/PI, NH	Latina	*P* value
N (%)	39 036 (47.6%)^a^	20 489 (25.0%)^a^	7595 (9.3%)^a^	14 882 (18.1%)^a^	
Age group					
<50	7413 (19.0%)	5122 (25.0%)	2778 (36.6%)	3951 (26.6%)	<.0001
50‐74	21 670 (55.5%)	11904 (58.1%)	4189 (55.2%)	8651 (58.1%)	
75+	9953 (25.5%)	3463 (16.9%)	628 (8.3%)	2280 (15.3%)	
Nativity					
US born	19 185 (49.2%)	9064 (44.2%)	306 (4.0%)	2150 (14.5%)	<.0001
Foreign born	7508 (19.2%)	5228 (25.5%)	5327 (70.1%)	7449 (50.1%)	
Unknown	12 343 (31.6%)	6197 (30.3%)	1962 (25.8%)	5283 (35.5%)	
Poverty level^b^					
0%‐<5%	8534 (21.9%)	1659 (8.1%)	959 (12.6%)	988 (6.6%)	<.0001
5%‐<10%	12 238 (31.4%)	2766 (13.5%)	1471 (19.4%)	1608 (10.8%)	
10%‐<20%	11 725 (30.0%)	5336 (26.0%)	2749 (36.2%)	3445 (23.2%)	
20%‐100%	6507 (16.8%)	10714 (52.3%)	2406 (31.7%)	8823 (59.3%)	
Unknown	32 (0.1%)	14 (0.1%)	10 (0.1%)	18 (0.1%)	
Stage					
Local	24 402 (62.5%)	10 898 (53.2%)	4784 (63.0%)	8573 (57.6%)	<.0001
Regional	10 522 (27.0%)	6904 (33.7%)	2258 (29.7%)	4925 (33.1%)	
Distant	2352 (6.0%)	1784 (8.7%)	322 (4.2%)	857 (5.8%)	
Unknown	1760 (4.5%)	903 (4.4%)	231 (3.0%)	527 (3.5%)	

Abbreviation: NH, Non‐Hispanic.

^a^ Row percent for age group, nativity and poverty level; column percent for stage.

^b^ Poverty level: the proportion of the neighborhood living at or below the federal poverty level.

**FIGURE 2:**
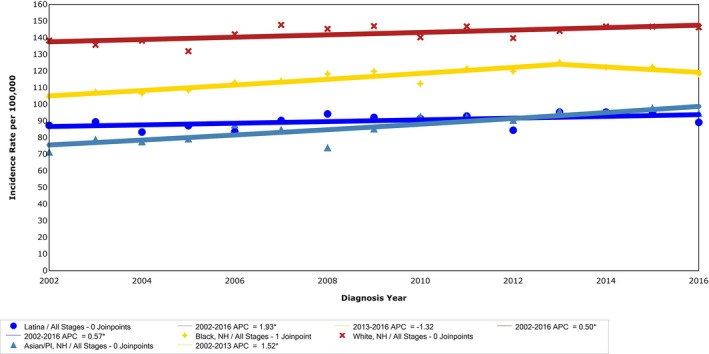
Incidence rate of breast cancer among women in NYC by race/ethnicity, 2002‐2016. Joinpoint analysis of trends in NYC women breast cancer incidence rates per 100 000 population by race/ethnicity, from the NYSCR 2002‐2016. APC, annual percent change; NYSCR, New York State Cancer Registry

Between 2002 and 2016, breast cancer incidence increased by 21.1%. The annual change in incidence rate increased minimally (APC 0.6, 95% CI 0.3, 0.8). Comparison of average annual incidence and APC by race/ethnicity group is presented in Table 2. White women had the highest overall average annual incidence of breast cancer (142.5/100 000), followed by Black women (115.6/100 000), Latina women (90.1/100 000) and Asian/Pacific Islander women (86.6/100 000). The relative increase in incidence rate from 2002 to 2016 was 33% for Asian/Pacific‐Islander women, 13% for Black women, 9% for Latina women and 6% for White women. Pairwise comparison demonstrated that Asian/Pacific‐Islander women had a significantly higher rate of increase in breast cancer incidence rate between 2002 and 2016 (APC = 1.9) than any other race/ethnicity. The rate of change was higher among Black women as compared to White women (*P* < .01) (Figure 2).

**TABLE 2:** Average annual breast cancer incidence and mortality rate and percent change by race/ethnicity among women in NYC, 2002‐2016

	White, NH	Black, NH	Asian/PI, NH	Latina
	(N = 39 036)	(N = 20 489)	(N = 7595)	(N = 14 882)
Incidence rate				
Rate (mean, SD)	**142.5 (4.9)** ^b^, ^c^, ^d^	**115.6 (6.6)** ^a^, ^c^, ^d^	**86.6 (8.6)** ^a^, ^b^	**90.1 (4.3)** ^a^, ^b^
APC (95% CI)	**0.5 (0.2, 0.9)** ^b^, ^c^, ^d^	**0.9 (1.3, 2.6)** ^a^, ^c^, ^d^	**1.9 (1.3, 2.6)** ^a^, ^b^	**0.6 (0.0, 1.1)** ^a^, ^b^
Mortality rate				
Rate (mean, SD)	**23.8 (2.8)** ^b^, ^c^, ^d^	**28.3 (2.4)** ^a^, ^c^, ^d^	**9.7 (1.6)** ^a^, ^d^	**16.0 (1.4)** ^a^, ^c^
APC (95% CI)	**−2.3 (−3.0, −1.6)** ^b^, ^c^, ^d^	**−1.5 (−2.2, −0.7)** ^a^, ^c^, ^d^	−0.2 (−2.4, 2.0)^a^, ^b^, ^d^, ^1^	**−1.2 (−2.1, −0.3)** ^a^, ^b^, ^c^
Mortality‐to‐incidence ratio				
Rate (mean, SD)	**0.17 (0.02)** ^b^, ^c^, ^d^	**0.25 (0.03)** ^a^, ^c^, ^d^	**0.11 (0.02)** ^a^, ^d^	**0.18 (0.02)** ^a^, ^c^
APC (95% CI)	**−2.8 (−3.6, −2.0)** ^b^, ^c^, ^d^	**−2.6 (−3.4, −1.7)** ^a^, ^c^, ^d^	**−2.3 (−4.4, −0.3)** ^a^, ^b^, ^d^	**−1.7 (−2.8, −0.6)** ^a^, ^b^, ^c^

^a^ Significantly different from White at *α* = 0.05.

^b^ Significantly different from Black at *α* = 0.05.

^c^ Significantly different from Asian/PI at *α* = 0.05.

^d^ Significantly different from Latina at *α* = 0.05.

^1^ Unbolded APC values Asian/PI, NH means AAPC trend not significantly different from zero at *α* = 0.05.

Comparisons of stage at diagnosis by race/ethnicity are presented in Table 1. Pairwise sub‐analysis of distribution of stage at diagnosis by race/ethnicity group demonstrated significant differences on all analyses (*P* < .0001). White and Asian/Pacific‐Islander women had a significantly higher rate of local stage disease (Stage I) than Latina women, which were all also significantly higher than Black women with the lowest rate of local stage disease (*P* < .0001). Although percent of cases with local disease among Black women increased by 33%, their rate in 2016 (65.0%) remained lower than that of White women in 2002 (80.8%) (data not shown). Across the entire study period, Black women had a significantly higher rate of distant disease (Stage IV) than other groups; 33% higher than White and Latina women and 56% higher than Asian/Pacific‐Islander women (*P* < .0001).

**FIGURE 3:**
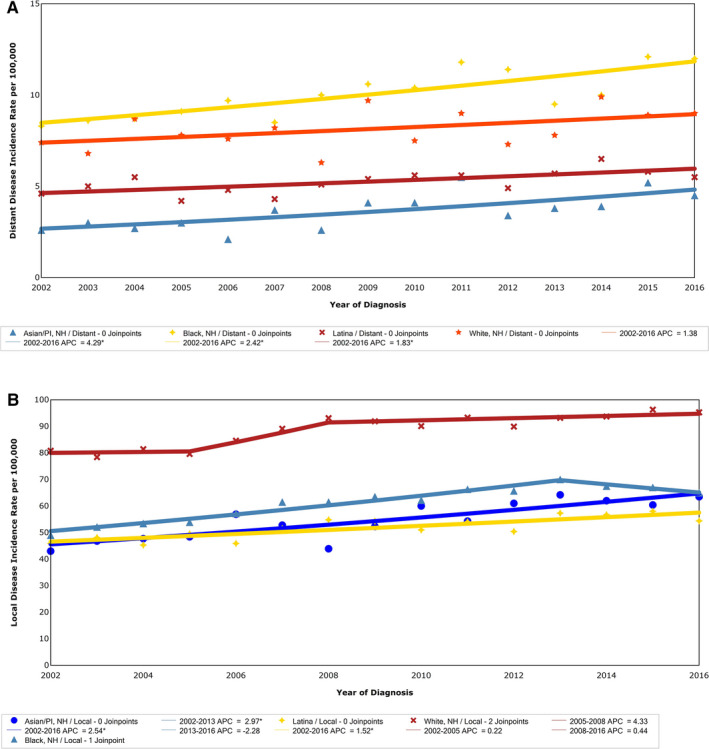
A, Percent of women with distant stage of breast cancer in NYC by race/ethnicity, 2002‐2016. Joinpoint analysis of trends in NYC women distant stage breast cancer incidence per 100 000 population by race/ethnicity, from the NYSCR 2002‐2016. B, Percent of women with local stage of breast cancer in NYC by race/ethnicity, 2002‐2016. Joinpoint analysis of trends in NYC women local stage breast cancer incidence per 100 000 population by race/ethnicity, from the NYSCR 2002‐2016. APC, annual percent change; NYC, New York City; NYSCR, New York State Cancer Registry

Trend analysis of the rate of distant disease at diagnosis demonstrated a significant increase among Asian, Black, and Latina women but not White women across the study period. However, these were not significantly different among race/ethnicity groups (Figure 3A). Similarly, all race/ethnicity groups except for White women had a significant increase in the rate of percent of cases diagnosed with local disease; however, the rate of change was not different between groups (Figure 3B).

### 3.3 Mortality and race/ethnicity

**FIGURE 4:**
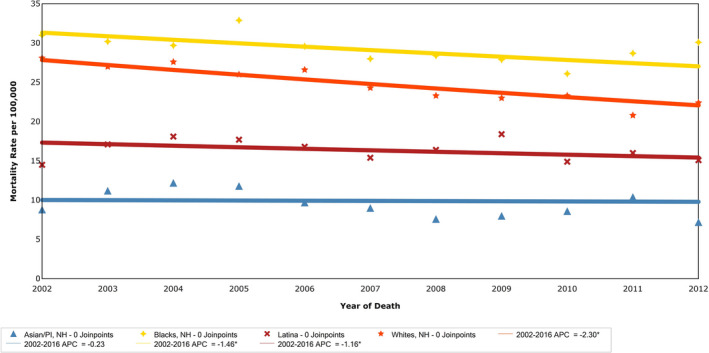
Mortality rate of breast cancer among women in NYC by race/ethnicity, 2002‐2016. Trends in NYC women age‐adjusted breast cancer mortality per 100 000 by race/ethnicity, NYSCR 2002‐2016. APC, annual percent change; NYC, New York City; NYSCR, New York State Cancer Registry

There were 16 225 breast cancer‐related mortalities in NYC during the study period. The breast cancer related mortality rate decreased by 17.1% from 2002 to 2016. Between 2002 and 2016, the mortality rate among White women decreased by 17.8% (from 28.1 to 23.1) as compared to a decrease of 15.5% (from 31 to 26.2) among Black women, Figure 4. Mortality rate decreased at APC 1.16 among Latina women by 2.8% (from 14.5 to 14.1) while mortality rates among Asian/Pacific‐Islander women remained relatively constant over the same time period.

The average decrease in mortality rate was 2.3% per year (APC −2.3, CI −2.8, −1.9) across the study period for women of all race/ethnicities. Age‐adjusted breast cancer mortality by race/ethnicity is presented in Figure 4. Black women had the highest mortality across all study years as compared to other race/ethnicity groups. (Table 2 and Figure 4) White women had the largest decline in annual rate of change in mortality rate (APC −2.3, 95% CI −3.0, −1.6, Figure 4). Pairwise comparison demonstrated that the annual rate of change in mortality rate was significantly greater for White women as compared to each other race/ethnicity group (*P* < .05). The mortality rate did not decline among Asian women. However, pairwise comparison among Black, Latina, and Asian/Pacific Islander women found no significant differences between groups.

### 3.4 Mortality‐to‐incidence ratio

**FIGURE 5:**
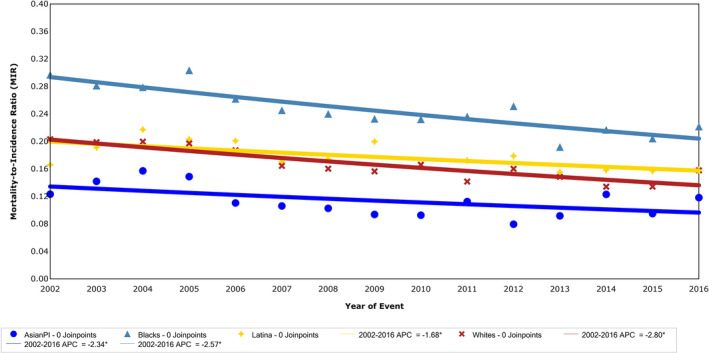
Joinpoint analysis of trends in MIR among NYC women with breast cancer, NYSCR 2002‐2016. APC, annual percent change; NYC, New York City; NYSCR, New York State Cancer Registry. Mortality‐to‐incidence ratio (MIR) among women in NYC by race/ethnicity, 2002‐2016

MIR decreased from 2002 to 2016 by about 3% annually (APC −2.7, CI −3.5, −2.0) overall for women of all race/ethnicities. The MIR across study years by race/ethnicity is presented in Figure 5. White women had a significantly faster rate of decrease than all other groups (*P* < .05). In 2002, the MIR among Black women was 1.5 times higher than White women (0.30 vs 0.20), and it remained 1.5 times higher 14 years later in 2016 among Black women as compared to White women (0.22 vs 0.16).

## 4 DISCUSSION

In this study of female breast cancer in NYC, we demonstrated that the MIR among White women was nearly half that of Black women. Despite higher mammography rates, Black and Latina women in NYC were less likely to be diagnosed with local stage breast cancer as compared to White and Asian/Pacific‐Islander women. Further, despite higher incidence of disease among White women, their mortality was lower than that of Black women. Mortality among White women decreased at a significantly faster rate than among Black women, and among Latina women mortality was stable. The observed differences mirror well documented racial/ethnic disparities in national female breast cancer mortality in the US.^6^, ^7^, ^8^ In addition, Black, Asian/Pacific Islander, and Latina women with breast cancer were younger and more likely to be living in higher poverty neighborhoods compared with White women.

This study presents an enhanced understanding of breast cancer inequities in NYC by looking at more racial/ethnic subgroups, the use of MIR, and putting the inequities in the context of screening trends. To our knowledge, this is the only study to include Latina and Asian/Pacific Islander groups or to use MIR to evaluate racial/ethnic disparities in breast cancer outcomes over time in NYC. This is important because it is known that Black and Latina women have a higher prevalence of triple negative disease than White women, which is associated with more aggressive pathology, and few studies have included Asian/Pacific‐Islander women.^15^, ^16^, ^17^, ^18^, ^19^, ^20^, ^28^ Further, including disease stage and the mortality‐incidence ratio provide better context to interpret the implications of differential incidence and mortality rates. One of our most striking findings is that our data suggest that earlier diagnosis and decreasing mortality are occurring in Black women over time. Yet, for both rate of early stage breast cancer and MIR, the current rates for Black women reflect where White women were more than a decade earlier. As breast cancer's amenability to treatment increases, continued monitoring and vigilance regarding these disparities is important as access to treatment options may not be equitably distributed.

Similar to national trends, this study demonstrated that the incidence gap between White and non‐White women decreased between 2002 and 2016.^29^ Although this may primarily reflect improved access to screening among all subpopulations, other important factors may also have a role in the observed gap. For example, the rise in breast cancer incidence among Black and Latina women has paralleled rising rates in obesity, a known risk factor for breast cancer. In NYC in 2010, obesity rates among Black and Latina women were nearly four times higher than those of White and Asian/Pacific‐Islander women.^23^

Changing incidence rates may have an important, and potentially modifiable, role in explaining observed differences in MIR. Our study demonstrated two worrisome trends in stage at diagnosis despite similarities in mammography rates during the study period. First, although the rate of women with local disease at diagnosis increased over the study period for all race/ethnicity groups, there was no difference in the rate of increase between groups. The second observation is that Black women had a significantly higher rate of distant disease at diagnosis than all other groups. A previous comparison of breast cancer incidence by race/ethnicity group and neighborhood income level in NYC demonstrated that Black women had a higher likelihood of late stage breast cancer at diagnosis compared to Latina women regardless of neighborhood, but that both groups had a higher risk than White women regardless of income.^30^ These findings are interesting given that rates of mammography in the past 2 years were relatively high among Black women. While this may be partially explained by differences in mammogram screening quality, or limitations in local survey methodology, which ask about the most recent mammogram and not screening intervals over longer periods of time or term frequency of mammography, further study is needed.^31^, ^32^ In a study of over a million women in the US between 1996 and 2002, Black women, but not Latina women, were less likely to have received adequate mammogram screening at recommended intervals; after controlling for screening interval, observed disparities in tumor stage and size were eliminated.^31^ In the current study, the possibilities of inadequate timing and quality of mammogram screening, differences in tumor biology, and/or disparities in evidence‐based treatment selection may all be important areas of investigation to explain the slower rate of decrease in mortality rate among Black women and stable MIR over time among Latina women. It is possible that even if there are differences in tumor biology, patient and provider education on recommended screening intervals may increase earlier diagnosis among women in NYC, especially Black women who have a higher mortality burden.

### 4.1 Limitations

Although this large cohort study of racial/ethnic trends in breast cancer incidence and mortality in NYC demonstrated differences in stage at diagnosis that may contribute to deviations in mortality rates, MIR analysis does not allow for any definitive conclusions as to the etiology of the observed mortality or changes in mortality rates. Furthermore, it is not possible with our data to control for the effects of tumor biology, disease duration nor health insurance status, which inevitably impacts quality of care received. Although tumor biology may have a role in the differences in MIR, racial/ethnic disparities in mortality rates have also been demonstrated to vary in rural South Carolina^12^ and between different cities, suggesting that differences in local health system factors such as screening and treatment may play a larger part in the poorer outcomes for Black women.^15^, ^18^, ^33^ Finally, although comparison of MIR does not identify factors associated with observed differences in outcomes between race/ethnic groups, the results of this study do illustrate that in a large, diverse population there are significant differences in outcomes that should be further addressed.

## 5 CONCLUSIONS

This study demonstrated that White and Asian/Pacific‐Islander women had a significantly lower breast cancer mortality burden than Black and Latina women, as defined by MIR. The decreasing incidence gap between White, Latina, and Black women, coupled with the greater decrease in mortality rate among White women, raises concerns about whether there is unequal access to high‐quality early stage diagnosis and treatment, by which Black women in NYC may be disproportionately impacted. Additionally, understanding how socioeconomic factors such as poverty and structural racism contribute to and intersect with these disparities is critical.^31^, ^32^, ^34^, ^35^ Study findings highlight the need for qualitative research to explain the troubling disparities, and more attention by health systems and physicians to devote resources to ensure they are addressed on a system level. Assessment of racial/ethnic differences in MIR in other local regions may help identify high‐risk healthcare systems or populations for targeted implementation of interventions that may improve equity in breast cancer outcomes.

## CONFLICT OF INTEREST

The authors have no relevant conflicts of interest to disclose.

## AUTHOR CONTRIBUTIONS

Tamar B. Nobel: Conceptualization, formal analysis, visualization, writing ‐ original draft. Charles K. Asumeng: Formal analysis, data curation, methodology, visualization. John Jasek: Conceptualization, methodology, writing ‐ review and editing. Kellie C. Van Beck: Conceptualization, writing ‐ review and editing. Ruchi Mathur: Conceptualization, formal analysis. Baozhen Qiao: Data curation, methodology. Jennifer J. Brown: Conceptualization, supervision, writing ‐ review and editing.

## Data Availability

All data are available from the corresponding author upon request.
